# Pharmacogenetics in Italy: current landscape and future prospects

**DOI:** 10.1186/s40246-024-00612-w

**Published:** 2024-07-10

**Authors:** Matteo Floris, Antonino Moschella, Myriam Alcalay, Annalaura Montella, Matilde Tirelli, Laura Fontana, Maria Laura Idda, Pharmacogenomics Working Group of the Italian Society of Human Genetics (SIGU), Paolo Guarnieri, Mario Capasso, Corrado Mammì, Paola Nicoletti, Monica Miozzo

**Affiliations:** 1 Department of Biomedical Sciences, University of Sassari, Sassari, Italy; 2 Unit of Medical Genetics, Grande Ospedale Metropolitano Bianchi-Melacrino-Morelli, Reggio Calabria, Italy; 3 Department of Experimental Oncology, European Institute of Oncology IRCCS, Milano, Italy; 4 Department of Oncology and Hemato-Oncology, University of Milan, Milano, Italy; 5 CEINGE Biotecnologie Avanzate, Napoli, Italy; 6 Department of Molecular Medicine and Medical Biotechnology (DMMBM), Università degli Studi di Napoli “Federico II”, Napoli, Italy; 7 Medical Genetics Unit, Department of Health Sciences, ASST Santi Paolo e Carlo, Università degli Studi di Milano, Milan, Italy; 8 23andMe Inc., Therapeutics, South San Francisco, CA USA; 9 Department of Genetics and Genomic Sciences, Icahn School of Medicine at Mount Sinai, New York, NY USA

**Keywords:** Pharmacogenetics, Pharmacogenomics, Germline testing

## Abstract

Pharmacogenetics investigates sequence of genes that affect drug response, enabling personalized medication. This approach reduces drug-induced adverse reactions and improves clinical effectiveness, making it a crucial consideration for personalized medical care. Numerous guidelines, drawn by global consortia and scientific organizations, codify genotype-driven administration for over 120 active substances. As the scientific community acknowledges the benefits of genotype-tailored therapy over traditionally agnostic drug administration, the push for its implementation into Italian healthcare system is gaining momentum. This evolution is influenced by several factors, including the improved access to patient genotypes, the sequencing costs decrease, the growing of large-scale genetic studies, the rising popularity of direct-to-consumer pharmacogenetic tests, and the continuous improvement of pharmacogenetic guidelines. Since EMA (European Medicines Agency) and AIFA (Italian Medicines Agency) provide genotype information on drug leaflet without clear and explicit clinical indications for gene testing, the regulation of pharmacogenetic testing is a pressing matter in Italy. In this manuscript, we have reviewed how to overcome the obstacles in implementing pharmacogenetic testing in the clinical practice of the Italian healthcare system. Our particular emphasis has been on germline testing, given the absence of well-defined national directives in contrast to somatic pharmacogenetics.

**Supplementary Information:**

The online version contains supplementary material available at 10.1186/s40246-024-00612-w.

## Background

Since the late 1950s, investigators [1–3] have documented the connection between genetic background and response to medications as well as the importance of maximizing the benefits and minimizing the harm of treatments. As more pharmacogenetics (PGt) evidence accumulated over the decades, a “genotype-tailored” approach to pharmacological therapy emerged, whose advantages extend not only to the patients but also have significant implications for drug developers and National Health Systems (NHSs). However, in certain regions, implementation of PGt still needs to be consolidated.

In general, two different types of PGt testing are crucial to provide patients with the most effective treatments: *somatic testing*, used for tailored anti-cancer therapies, and *germline testing*, useful in predicting individual drug responses based on inherited genetic variations.

Somatic PGt testing is commonly employed in oncology to predict the efficacy of cancer treatments or drug resistance; examples are  *EGFR* mutations and response to tyrosine kinase inhibitors [3]; *BRCA1/2* variants and PARP inhibitors [4]; *KRAS/NRAS/BRAF* mutations and *cetuximab* resistance [5]. In Italy, molecular testing in oncology is standardized by the *Italian Association of Medical Oncology* (AIOM) in collaboration with The *Italian Society of Pharmacology* (SIF), which promote guidelines for cancer therapy in different clinical scenarios [6].

Germline PGt testing can be used in two different frameworks: i) the evaluation of potential benefits of a treatment in presence of a specific genotype, as exemplified by the use of *lexacaftor/ ivacaftor/ tezacaftor*, prescribed for cystic fibrosis (CF) therapy only in patients with at least one copy of the F508del mutation (NM_000492.4:c.1521_1523del) in the *CFTR* gene [7]); ii) the investigation of functional genetic variants influencing the pharmacokinetics (PK) and/or the pharmacodynamics (PD) of a drug, thereby affecting the drug’s efficacy, appropriate dosage, and adverse effects.

Pharmacokinetics (PK) genetic variants influence drug absorption, liver metabolism, distribution, and excretion (ADME genes); a key role of *CYP2D6* -- a highly polymorphic gene involved in the metabolism of up to 25% of the approved drugs – has been clearly demonstreated in PK regulation [8–13]. One of the most remarkable examples is *siponimod*, a drug prescribed for secondary progressive multiple sclerosis (SPMS) whose dosage must be adapted after genetic testing to maximize efficacy. The drug is metabolized in the liver by the polymorphic pharmacogene *CYP2C9*; accordingly, both the *Food and Drug Administration* (FDA) and *European Medicinal Agency* (EMA) require *CYP2C9* genotyping before treatment with *siponimod* [14, 15]. In Italy, an official AIFA note refers to genetic testing for prescribing *siponimod*: “[…] Before starting treatment, it is necessary to determine the *CYP2C9* genotype of patients with the aim of establishing their *CYP2C9* metabolizer status […]. In patients homozygous for the allele CYP2C9*3 (NG_008385.2:g.48139A > C), siponimod should not be used” [16]. However, the Italian version of the leaflet of the drug *Mayzent* (commercial name of Siponimod) makes no mention of these indications, although the test is required by the ‘Summary of Product Characteristics’ also released by AIFA. [17].

Pharmacodynamics (PD) genetic variants, on the other hand, may influence the interaction between the active drug and effector molecules. An illuminating case is represented by the potent broad-spectrum aminoglycoside antibiotics, often used for the treatment of suspected infections in neonatal intensive care units (NICU): aminoglycoside-induced hearing loss (AIHL) is associated with at least three variants in the *MT-RNR1* gene (m.1095T > C, m.1494 C > T, and m.1555 A > G) [18].

In this manuscript, we focus on the germline PGt tests, which are currently applied to a limited number of drugs within in the Italian healthcare system.

### Factors driving the introduction of pharmacogenetics in healthcare systems

Many primary factors drive the implementation of pharmacogenetic testing within NHSs.

The cost reduction of genotyping and sequencing technologies has led to the widespread adoption of genetic testing across various domains, both clinical and direct-to-consumer.

Moreover, there is a mounting body of evidence demonstrating the clinical utility of validated germline pharmacogenetic testing [19]. An interesting analysis carried out across 15 US institutions provided an overview of current efforts to implement a preventive (*pre-emptive*) PGt testing strategy in clinical practice aiming for the “*Right Drug, Right Dose, Right Time*” approach [20, 21]. The genes pre-emptively tested varied among sites, but generally included *CYP2C19, CYP2C9, VKORC1*, and *CYP2D6.* These genes were consistently analyzed prior the prescription of selective *serotonin reuptake inhibitors* (SSRIs), *voriconazole*, *clopidogrel*, *opioids*, and *warfarin*.

The clinical utility of a *pre-emptive* strategy was further demonstrated by the *Pre-emptive Pharmacogenomic Testing for Preventing Adverse Drug Reactions* (PREPARE) trial, a recent multi-center, controlled, cluster-randomized study [19] in which a 12-gene PGt panel was used to accurately genotype the selected pharmacogenes in 18 hospitals, 9 community health centers and 28 community pharmacies in seven European countries. Italy was one of the partner countries. Participants were genotyped for 50 germline variants, and those with an *“actionable”* variant were treated according to the *Dutch Pharmacogenetics Implementation Working Group* (DPWG) recommendations, whilst patients in the control group received standard treatment. The results indicated a 30% reduced risk of clinically relevant adverse reaction (OR = 0.70 [95% CI 0.54–0.91]; *p* = 0.0075), demonstrating that genotype-guided treatment significantly reduced the incidence of clinically relevant adverse drug reaction (ADR) and is feasible in different organizations and health system settings. This kind of approach is based on multiple genotyping which may be useful also for guiding future treatments.

The rapid spread of genetic biobanks is also driving the growth of pharmacogenetics; indeed increasing amount of data require the adoption of recommendations by the NHSs [22]. Population-scale studies revealed that over 95% of the general population carries at least one actionable genotype or diplotype [23, 24]. On the other side, half of all prescriptions in the United States is potentially affected by actionable germline PGt variants [25]. With the advent of population-scale research initiatives, it is also becoming feasible to estimate the prevalence of the most relevant pharmacogenetic variants in the European [26] and Italian populations [24, 27]. Therefore, it is this becoming possible to assess which part of the population may be targeted by preventive pharmacogenetic initiatives.

Furthermore, with a view to a cost efficiency, it would be interesting to consider taking advantage of routine comprehensive genetic tests performed using NGS analysis for other purposes (such as exome sequencing or targeted panels) to extract pharmacogenetic information for benefit the individual. This approach would provide a clear cost saving for the analysis, bearing in mind that in most cases these subjects require chronic drug therapies. Recent studies have investigated the feasibility of identifying incidental findings, namely pathogenic or likely pathogenic variants in pharmacogenes, as a secondary finding of NGS analyses initially performed for other diagnostic purposes [28–31]. However, currently, there are no specific recommendations from the *American College of Medical Genetics* (ACMG) regarding this topic, and the interpretation of such variants remains challenging. This complexity arises from the high probability of detecting incidental finding and the fact that, unlike disease-related genes, the influence of drug-related genes is often modulated by environmental factors. According to the ACMG recommendations for incidental findings from WES/WGS analyses reporting [32], Malignant Hyperthermia Susceptibility (OMIM #145,600) is the sole condition associated with genes included in the *Clinical Pharmacogenetics Implementation Consortium* (CPIC) guidelines, for which the use of volatile anesthetics and *succinylcholine* should consider *RYR1* and *CACNA1S* genotypes. Consequently, it is advisable to report the presence of pathogenic or likely pathogenic variants in these two genes as a secondary outcome [33].

Finally, a phenomenon that cannot be ignored is the market of “direct-to-consumer genetic testing” (DTC-GT). In this regard, the company *23andMe*, which has more than 14 million customers worldwide [34], provides an FDA-approved pharmacogenetic report based on the genotype of *CYP2C19*, *DPYD*, and *SLCO1B1* genes.

### Pharmacogenetic guidelines

Best practices and algorithms to assist the choice of therapy according to genotype are codified by guidelines formulated by international consortia and scientific societies, edited by groups of experts, and usually published in scientific journals.

According to PharmGKB [35] – the primary pharmacogenomic knowledge-based resource created to aggregate all clinically relevant pharmacogenetic information -- there are currently 260 clinical guidelines for 194 active substances and combinations, published by different networks and scientific societies, including CPIC [36], DPWG [37], and other scientific societies, including the *Canadian Pharmacogenomics Network for Drug Safety* (CPNDS) [38] and the *French National Network of Pharmacogenetics* (RNPGx) [39], which has around 30 members throughout France and other French-speaking countries (Belgium and, more recently, Switzerland and Canada). Guidelines are also established in other countries, including Germany and Spain.

Among the 291 variant-drug combinations categorized in PharmGKB [40] as “Level 1A” (accessed on 15 Jan 2024) --indicating inclusion in at least one major clinical guideline -- half are associated with genetic variants that elevate the risk of drug toxicity. The remaining 50% concerned variants affecting efficacy (17%), dosage (5%), drug metabolism (24%) or combinations of these mechanisms. In addition, 106 combinations are of interest in paediatric care settings.

CPIC guidelines were created in 2009 to provide dosing guidance related to PGx information present in the medical record, to assist healthcare providers in making informed decisions based on genetic testing results [41, 42]. In a scenario where preventive and clinical genotyping are becoming increasingly common and accessible, CPIC guidelines represent a key resource.

Currently, CPIC [43] has published level A or B guidelines for of 110 gene/drug pairs, mainly regarding cytochromes *CYP2D6* (16% of gene/drug pairs), *CYP2C19* (13%) and *CYP2C9* (10%); the most frequently found gene after the 3 cytochromes is *MT-RNR1* (10%) [Suppl. Table “CPIC (A and B)”]. A total of 85 drugs are considered in these guidelines.

According to PharmGKB [Suppl. Table “PharmGKB guidelines (w. rec)”], and excluding the drugs with a “*no recommendation*” statement (e.g., the case of *aspirin*, for which CPIC states “*CYP2C9*: no recommendation; *G6PD*: no recommendation”), there are 107 active substances for which at least one guideline provides advice for alternate drug or change in dosage: 80 active substances are covered in the CPIC guidelines; 54 in DPWG guidelines; 23 in other guidelines.

Furthermore, guidelines from the CPIC cover three-quarters of drugs; among these, 46 active ingredients are not currently considered by guidelines from other scientific societies or consortia guidelines. Moreover, there are 27 drugs for which no CPIC guidelines exist.

Interestingly, for 5 of the 20 most widely consumed active ingredients in Italy [44], CPIC claims that there is sufficient evidence to recommend at least one prescriptive action (CPIC level A and B gene/drug pairs), i.e.: *pantoprazole*, *lansoprazole*, *omeprazole* (*CYP2C19*), with “*Actionable PGx*” indications in the FDA labels and “*Moderate/Optional*” CPIC strength of evidence; *atorvastatin* (*SLCO1B1*), with “*Informative PGx*” in the FDA label, with “*Moderate*” CPIC strength of evidence; *rosuvastatin* (*ABCG2* and *SLCO1B1*), with “*Actionable PGx*” in the FDA label, with “*Strong type*” CPIC strength of evidence for *SLCO1B1* and “*Strong/Moderate*” type for *ABCG2*.

Several studies have explored the appropriateness of patients genotyping to improve patient outcomes and prevent the discontinuation of treatment due to either ineffectiveness and/or the occurrence of side effects. These issues indirectly increase the costs for the Italian NHS. For instance, a recent study developed a cost-effectiveness model for the introduction of multiple pharmacogenetic tests (*CYP2C19, CYP2C9, CYP4F2, VKORC1*) in a hypothetical cohort of patients with acute coronary syndrome and/or atrial fibrillation, supported by electronic and informatics tools. This approach has suggested a real improvement in patient quality of life of, along with a reduction in clinical events and costs for the NHS [45]. Of note, this approach considered drug efficacy, which is often excluded from assessment of the potential impact of PGt testing. Future research could benefit from integrating these endpoints for a better characterization of pre-emptive PGt tests.

The DPWG was founded in 2005 by the Royal Dutch Pharmacist’s Association (KNMP).

The DPWG’s recommendations are available on the KNMP website (in Dutch) and are updated periodically [46–48]. According to PharmGKB reports, the DPWG guidelines include recommendations for genetic testing for 54 active substances. Among these, 18 active substances are exclusive to the DPWG guidelines and not mentioned in other guidelines [Suppl. Table “ST PharmGKB guidelines (w. rec)”]. These recommendations have been instrumental in selecting actionable drug–gene interactions for the PREPARE study [19]. The implementation of this panel, known as the “*PGx-Passport*”, which include the most prevalent genes among the DPWG annotation (*CYP2B6, CYP2C9, CYP2C19, CYP2D6, CYP3A5, DPYD, F5, HLA-B, SLCO1B1, TPMT, UGT1A1* and *VKORC1*), has been demonstrated to be adaptable to various European health-care systems.

### Privacy issues and prescription: the legal framework

In an era characterized by the rapid increase of databases containing large collections of human genomic data, the protection of personal information has become a critical issue. Genomic data represents a crucial resource for biomedical and clinical research, but protecting the privacy of personal data from illegal or unauthorized for-profit uses is increasingly challenging.

On May 25, 2018, the European Union (EU) regulation 2016/679 known as the General Data Protection Regulation (GDPR) came into force, abrogating all previous laws concerning data protection [49]. Under GDPR statute, the processing of special categories of personal data, including genetic data, should be allowed only when strictly necessary [“Article 10”]. Such processing must be preceded by the individual’s consent, which should be informed, complete and specific [“Article 13”]. For pharmacogenomic testing, the same general regulations apply. Hence, consent to process genetic data is always required and it would be desirable to specifically supplement the informed consents currently in use for diagnostic examinations.

In our opinion, with regard to the practice of testing, the prescription of the test could be requested by a specialist doctor or by a general practitioner, whilst the role of the geneticist should remain central in counselling, testing and interpretation of the result. This approach is particularly valuable when genetic testing is not contingent on an evaluation of family history or a risk calculation [50].

### Current status of pharmacogenetics guidance by EMA and pharmacogenetic testing prescriptions in the context of the Italian NHS

Although there are examples of the implementation of pharmacogenetic guidelines in european NHSs, a certain discordance exists between these national initiatives and the regulations set by the EMA. Furthermore, there is a lack of uniformity in the management of pharmacogenetic testing and clinical practices across various European countries.

According to PharmGKB (accessed on 15 Jan 2024), EMA has approved 95 drugs for which “*required*” or “*recommended*” pharmacogenetic indications are available in the respective labels. At the time of access, there were also 135 “*informative*” or 44 “*actionable*” label annotations. As the active substance *pegloticase* is currently withdrawn from the European market, the actual number is 94 drugs with indications “*required*” (*N* = 91) or “*recommended*” (*N* = 3) [Suppl. Table “ST Table EMA”].

In detail, in 26 out of 94 cases, the genetic test is germline (27.7%), in 7 of them it predicts an enzymatic activity (7.5%), while for the remaining active ingredients the test is for somatic mutations (64.9%) (Table 1). Most of the drugs considered (64/94, 68%) are classified as antineoplastic agents according to the EMA classification [Suppl Table “ST Table EMA”].

**Table 1:** Category of genetic test in the EMA labels (for drugs approved in the European market), grouped by the Human pharmacotherapeutic group (EMA) (extended details in Suppl Table “ST Table EMA”)

Pharmacotherapeutic group	Germinal	Other	Somatic	Withdrawn	Total
Antiepileptics, other antiepileptics	1				1
Antigout preparations				1	1
Antineoplastic agents (+ Monoclonal antibodies, Protein kinase inhibitors, immunomodulating agents)	6		58		64
Antiobesity preparations, excl. diet products	1				1
Antivirals for systemic use	2				2
Bile acids and derivatives		1			1
Endocrine therapy			1		1
Lipid modifying agents	1				1
Other alimentary tract and metabolism products	6	6	1		13
Other cardiac preparations	1				1
Other drugs for disorders of the musculo-skeletal system	1				1
Other hematological agents	1				1
Other respiratory system products	4				4
Other therapeutic radiopharmaceuticals			1		1
Rare disease	1				1
Selective immunosuppressants	1				1
*Grand Total*	26	7	61	1	95

The ‘germinal’ category includes those drugs for which germline genetic testing is intended. The ‘somatic’ category, on the other hand, includes those drugs for which genetic tests are performed on tissue, mainly in the contest of cancer therapy. In the category “other” we have included specific drugs for certain genetic mendelian disorders, such as inborn errors or enzymatic deficiencies, for which genetic or laboratory tests (e.g. biochemical assay or enzymatic activity) are required prior to intake, according to the EMA annotations reported on PharmGKB. In this case, the test is obviously performed prior to taking the drug, as the therapy is specific to the identified enzyme defect. The EMA annotations for this group of drugs generally refer to the diagnosis of the disease, which in this case is usually achieved by performing a biochemical assay. As a rule, it is only afterwards that genetic confirmation of the result is carried out. In the cases of 4 drugs for metabolic disease (*betaine, carglumic acid, fosdenopterin, migalastat*), on the other hand, diagnosis by genetic testing is known to be primary/obligatory or necessary to identify specific ‘*amenable*’ variants for therapeutic indications. Hence, these drugs are included in the “*germinal*” category. However, the analysis of these genes is intended for therapeutic purposes and not for dose modulation/prevention of adverse events, therefore it was not the object of this study

Considering the 20 non-antineoplastic drugs for which germline testing is required by EMA, in most of them the test is motivated by diagnostic purposes, and not to predict the efficacy or toxicity of the drug (e.g. drugs used for cystic fibrosis, such as *elexacaftor* and *ivacaftor*).

Currently, considering only drug/gene pairs for which EMA provides annotations for preventing adverse events and/or dose modulation, only the following genes are included: *HLA, CYP2C19, CYP2D6, CYP2C9* and *DPYD* [Suppl Table “ST Table EMA”].

To date, in Italy, the availability of molecular genetic tests for pharmacogenetic purposes has been paradoxically facilitated by unclear legislation generically referring to the EMA/AIFA recommendations. However, the publication of various decrees over the last 7 years has revealed certain contradictions and inconsistencies.

From a practical point of view, the list of medical prescriptions of any genetic test recognized by the Italian NHS is regulated through the *Essential Levels of Care* (LEA) framework. The LEAs consist of a comprehensive repository of treatments and services that is required to provide to all citizens, either free of charge or after payment of a participation fee (the so-called “ticket”).

Recently, the Italian government has updated this repository to better define the services provided and the corresponding cost. However, with regards to the implementation of pharmacogenetic testing, this novel decree [51, 52] has excluded most of the pharmacogenetic analyses already approved by EMA and some of the tests supplied so far.

The new fee schedule is based on a list of healthcare services approved in 2017, which included the analyses of *CYP2D6, CYP2C19* and *UGT1A1* genes, while excluded *CYP2C9* and *DPYD* despite they are both required to avoid adverse events during specific pharmacological therapies (i.e., *siponimod* and *capecitabine*, respectively [14, 53]). Moreover, the prescription of these three tests would also be subjected to very restrictive criteria, relating to only 3 drugs. Therefore, this update does not consider either a large number of tests that are regularly prescribed and performed nationwide (e.g., *DPYD, HLA* typing, etc.) or the most recent international recommendations regarding the implementation of new pharmacogenetic tests.

A key point is that this update will significantly reduce the public offer of genetic testing for patients undergoing therapies that could benefit from specific genotyping, effectively limiting the EMA’s recommendations. However, the effective date of application of the new LEAs has been postponed to the next year (1 January 2025) [54]; this paper therefore aims to raise the issue of a revision of the regulation before the rule becomes operative.

Oncological patients deserve special mention, because most of the actionable PGt variants are associated to anticancer drugs in different guidelines. The impact of PGt testing on this subpopulation of patients is consistent with a high rate of prescription, due to the high risk of severe adverse events following therapy. A critical example is that of *DPYD* genotyping. About 30% of patients undergoing chemotherapy with fluoropymirimidines have ADRs due to a decrease of the *DPYD* activity, though additional gene variants have been associated with the ADRs. Therefore, *DPYD* genotyping is widely recommended as a pre-emptive test and its exclusion from updated LEA will likely result in a health-related and legal problem for oncologists as the test is recommended by an official AIFA note of May 2020 [55].

In order to resolve these conflicting and divergent recommendations at the national and international level, following the example proposed in this paper, other European countries could also assess the overlap between national regulations, EMA indications and the most recent scientific evidence, such as reported in the PREPARE study. This could make easier to align the different European regulations and keep the recommendations provided by EMA up-to-date.

### Towards a full application of pharmacogenetic practices in the Italian NHS

The Pharmacogenetics Working Group of the Italian Society of Human Genetics (SIGU) aims to establish the groundwork for the standardization of pharmacogenetic practices within the Italian NHS. This initiative begins with the incorporation of international guidelines based on the pharmacogenetic panel outlined in the PREPARE study [19]. Additionally, the group aims to expand and clarify the existing EMA/AIFA germline tests recommendations in the framework of the new LEAs.

Of the 40 drugs in the PREPARE panel (*clozapine, efavirenz, carbamazepine, sertraline* and *oxycodone* were removed according to indications provided in Supplementary files of Swen et al.), each of them has a guideline from the DPWG. Additionally, 26 of these drugs (72%), also have a guideline from CPIC (Table 2).

**Table 2:** Full list of drugs included in the PREPARE study which are on the market in Italy with indication for PGt by at least CPIC

Drug class	Drug	Clinically relevant gene	CPIC	Other guidelines	EMA	AIFA category
Anticoagulation	clopidogrel	*CYP2C19*	1	1		Informative PGx
Antidepressant	citalopram	*CYP2C19*	1			
Antidepressant	escitalopram	*CYP2C19*	1			Informative PGx
Antidepressant (TCA)	clomipramine	*CYP2C19*	1			Informative PGx
Antidepressant (TCA)	imipramine	*CYP2C19*	1			
Anti-infective	voriconazole	*CYP2C19*	1	1		Informative PGx
Anticoagulation	warfarin	*CYP2C9*	1	1		
Antiepileptic	phenytoin	*CYP2C9*	1			Informative PGx
Antiarrhythmic	flecainide	*CYP2D6*				Informative PGx
Antiarrhythmic	propafenone	*CYP2D6*				Informative PGx
Analgesic	codeine	*CYP2D6*	1	1		Informative PGx
Analgesic	tramadol	*CYP2D6*	1			Informative PGx
Anticancer	tamoxifen	*CYP2D6*	1	1		Informative PGx
Antidepressant	paroxetine	*CYP2D6*	1			Informative PGx
Antidepressant	venlafaxine	*CYP2D6*	1			Informative PGx
Antidepressant (TCA)	amitriptyline	*CYP2D6*	1			
Antidepressant (TCA)	clomipramine	*CYP2D6*	1			Informative PGx
Antidepressant (TCA)	imipramine	*CYP2D6*	1			
Antidepressant (TCA)	nortriptyline	*CYP2D6*	1			Informative PGx
Antihypertensive	metoprolol	*CYP2D6*				Informative PGx
Antipsychotic	aripiprazole	*CYP2D6*				Informative PGx
Antipsychotic	haloperidol	*CYP2D6*				Informative PGx
Antipsychotic	pimozide	*CYP2D6*				Testing Recommended
Antipsychotic	zuclopenthixol	*CYP2D6*				Informative PGx
Psychostimulant	atomoxetine	*CYP2D6*	1			
Immunosuppressive	tacrolimus	*CYP3A5*	1	1		Informative PGx
Anticancer	capecitabine	*DPYD*	1	1	1	Testing Recommended
Anticancer	fluorouracil	*DPYD*	1	1		Testing Recommended
Anti-infective	flucloxacillin	*HLA B*				Testing Recommended
Cholesterol- lowering	atorvastatin	*SLCO1B1*	1			
Cholesterol- lowering	simvastatin	*SLCO1B1*	1	1		
Immunosuppressive	azathioprine	*TPMT*	1	1		
Immunosuppressive	mercaptopurine	*TPMT*	1	1		
Immunosuppressive	thioguanine	*TPMT*	1			
Anticancer	irinotecan	*UGT1A1*		1		
Anticoagulation	acenocoumarol	*VKORC1*	1	1		
Anticoagulation	warfarin	*VKORC1*	1	1		

*Legend* CPIC = CPIC report exist for the drug/gene pair indicated; Guidelines = other (non CPIC or DPWG) guidelines exist for this drug/gene pair; EMA = drug for which there is an indication of a test required’ or ‘recommended’ by EMA; AIFA Category = class of pharmacogenetic information available in the current Italian drug labels

**Fig. 1:**
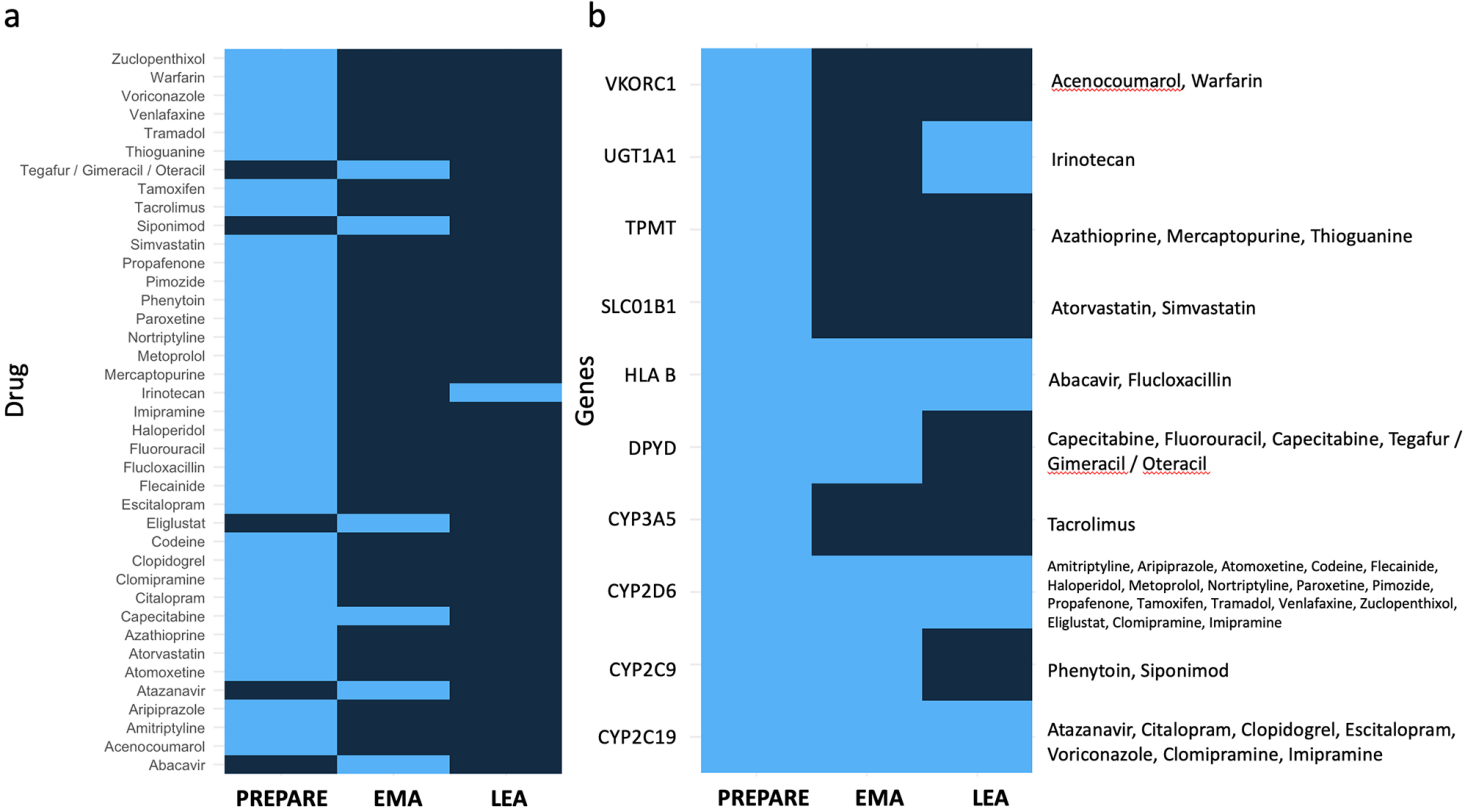
Comparison between drugs included in gene-drug pairs listed by Swen and colleagues (limited to drugs marketed in Italy) and drugs with label indications for germinal PGt tests according to EMA. Drugs or genes included by PREPARE, EMA and/or Italian LEA are coloured in light blue. With regard to EMA recommendations, only PGt tests with a modulating dose/AE prevention indication are considered, as explained in the main text. The left panel (**a**) shows the overlapping drugs between PREPARE and EMA lists. The right panel (**b**) compares the genes included into the PREPARE study with the genes noted by EMA recommendations and the Italian LEAs. Drugs associated with each gene with a pharmacogenetic indication are also shown in boxes. Considering the PGt tests included in the PREPARE panel that will be suitable in Italy after the implementation of the new LEAs, paradoxically the only test reported as recommended by the EMA (*DPYD*) will be excluded in the 2024 LEAs version. With regard to irinotecan, it has been included in the LEA column as the analysis of *UGT1A1* is scheduled, although the prescription of this test has not yet been fully clarified in the decree. Moreover, among the drugs reported by the EMA, only capecitabine overlaps with the PREPARE panel. It would therefore be useful to integrate the PREPARE list of drugs with that reported by EMA and to extend the genes noted in the new LEAs to those validated by Swen and colleagues

Currently, PGt testing “required” or “recommended” is indicated by EMA for predicting adverse events and/or dose modulation for six drugs (thus excluding all the therapeutic indications): *abacavir, atazanavir, capecitabine, eliglustat, siponimod, tegafur / gimeracil / oteracil* [Suppl Table “ST Table EMA”, genes and drugs highlighted in red]. However, by comparing the EMA gene-drug pairs with those included in the PREPARE panel (Table 2), only the *DPYD* genotyping for *capecitabine* administration is shared (Fig. 1).

Furthermore, regarding *DPYD*, EMA recommends a preventive test for the drug combination *tegafur / gimeracil / oteracil*, which is not included in the PREPARE panel. It would therefore be useful to extend this panel to drugs approved by AIFA that are analogous to those collected in the PREPARE study.

Another relevant issue concerns *siponimod*, for which *CYP2C9* genotyping is recommended by EMA. While the *CYP2C9* gene is included in the PREPARE panel for therapies based on *warfarin* and *phenytoin*, there is no specific indication for *siponimod*.

Regarding *CYP2D6* and *CYP2C19*, LEAs do not list drugs for which testing is appropriate, but rather provide a general indication for the pharmacogenetics of drug metabolism genes. Similarly, *irinotecan* is not directly mentioned in the LEAs, even though the analysis of known *UGT1A1* mutations (which are of oncological pharmacogenetic interest) is specified in the 2024 version.

Concerning *HLA* genotyping, the PREPARE panel includes only the *HLA-B* testing for the drug *flucloxacillin*, while the EMA annotations also indicate the genotyping of other HLA genes (for *abacavir*). According to the new LEAs, however, it is in any case allowed to prescribe all these tests as different MHC genes are listed and specified in detail. However, no indications are mentioned for using the test for pharmacogenetic purposes.

Moreover, 22 drugs approved by AIFA and considered in the PREPARE trial contain pharmacogenetic indications in the respective drug labels [Suppl. Table “AIFA extended”]. In more details, the drug labels of only three drugs recommend genetic testing prior to administration (Table 2): the anticancers *fluorouracil* and *capecitabine*, for which genotypic and phenotypic testing of *DPYD* is suggested; and the antipsychotic *pimozide*, for which testing of the *CYP2D6* gene is recommended to identify slow metabolizers. However, numerous efforts are underway to adapt the new decree to the local and national landscape by allowing the incorporation of certain adjustments to ensure an adequate implementation of pharmacogenetic testing.

It is of extreme interest to note that for several drugs (Table 2) -- some of which are widely used in Italy -- there is no indication of pharmacogenetic testing in AIFA-approved labels.

Striking examples are *citalopram* and *escitalopram*, for which the DPWG issued therapeutic dose recommendations based on *CYP2C19* genotype. For *CYP2C19* ultrarapid metabolizers, the recommendation is to avoid *escitalopram*, the most widely used antidepressant in Italy (OSMED Report2022).

To estimate the potential impact on the Italian NHS, it would be useful to consider data prevalence of pharmacogenetic variants in the Italian population and drugs prescription, but this has so far only been possible for certain subpopulations [24, 56].

In this regard, an Italian collaborative effort is ongoing to create a reference database for genomic data in the Italian Population (http://nigdb.cineca.it/), that can be searched for allele and genotype frequencies in the main macroareas [57, 58].

According to the latest report of the *National Observatory on the Use of Medicines* (AIFA OsMed 2022) (published on 7 August 2023) [59], the Italian public territorial expenditure -- including medicines dispensed to patients under contract, i.e., at full or partial charge of the NHS (class A) -- was around EUR 12.5 billion, with cardiovascular drugs representing the therapeutic class with the highest expenditure and usage. Among the top 30 active ingredients consumed in Italy in 2022 there are *clopidogrel*, *atorvastatin* and *simvastatin*. These three drugs are included in the PREPARE panel in relation to specific actionable genotypes. However for *atorvastatin* and *simvastatin* the AIFA-approved leaflet (as opposed to the Summary of Product Characteristics published by AIFA) does not include the analysis of the *SLCO1B1* gene, which is suggested by several guidelines and scientific papers.

According to the OsMed 2022 report, within class A medications group, the active ingredients with the highest expenditure are *atorvastatin*, *pantoprazole* and *cholecalciferol*. Furthermore, *cholecalciferol*, *ramipril* and *atorvastatin* are the most consumed active ingredients. Notably, in 2022, *atorvastatin* had the most significant financial impact on the NHS, with an expenditure of EUR 276 million. This data supports the hypothesis of the introduction of pre-emptive testing for statins, given their substantial impact on the population and the goal of optimizing public expenditure in relation to the effectiveness of treatment for patients.

Among the other drugs included in the PREPARE panel, certain categories are particularly relevant in terms of both consumption and financial impact for the NHS, such as *vitamin K* antagonists (*warfarin* and *acenocoumarol*). Antidepressants are another significant category, with annual consumption consistently rising and total expenditures reaching approximately EUR 300 million, encompassing both Selective Serotonin Reuptake Inhibitors (SSRIs) and Serotonin and Norepinephrine Reuptake Inhibitors (SNRIs). In 2022, the consumption of antidepressants accounted for 3.5% of the total drugs in Italy, with *SSRIs* accounting for approximately 70% of consumption. Also worth mentioning is the group of cytostatic antineoplastic drugs - antimetabolites, comprising several active ingredients but including in particular *5-fluorouracil*, *capecitabine*, *thioguanine*, *mercaptopurine*. Only for the first two, as illustrated above, are there specific pharmacogenetic recommendations on the package leaflet relating to DPYD analysis, despite the recommendations in international guidelines being applicable to all drugs in this class. Expenditure for this class of drugs is around EUR 80 million (more details in Sect. 3.11, 3.17 and 3.30 of report OsMed 2022).

A crucial aspect is the concept of “*prescriptive appropriateness”*. According to the “*prescriptive appropriateness decree*” published in Italy in 2016 [60], the use of pharmacogenetic analyses is only recommended in case of EMA/AIFA indications. This decree laid down the conditions for the dispensability of genetic tests and is currently in contradiction with the new LEAs, which have further restricted these services, particularly with regard to pharmacogenetic tests. However, the *appropriateness decree* does not indicate the genes for which a particular pharmacogenetic test is appropriate, but only indicates the service that can be provided. Only disease-associated genes included in the Orphanet database are clearly listed in the decree.

With a view to implementing and improving the PREPARE panel in the Italian healthcare context, it would be useful to expand the table of gene-drug pairs to incorporate the annotations provided by EMA/AIFA. In addition, assessing the evidence for analogous drugs that were initially excluded from the PREPARE panel is essential. This is particularly crucial for certain drug classes, such as *rosuvastatin*, for which CPIC guidance is available, but DPWG guidelines are not.

The recent updates of LEAs ensure that reimbursement for pharmacogenetic tests related to *CYP2C19, CYP2D6*, and *UGT1A1* is guaranteed. In this context, it is feasible to prescribe these tests prior to the prescription of different drugs as indicated in PREPARE table. However, LEAs do not offer specific guidance on the appropriateness of conducting these analyses.

Another point that requires clarification pertains to *HLA* genotyping. While the new LEAs allow this type of analysis, they do not specify any pharmacogenetic indications. This underscores the need for further guidance and protocols in the use of *HLA* genotyping in the context of pharmacogenetics within the Italian NHS.

Moreover, the new LEAs refer to the approval of a previous decree in 2017 (G.U. Serie Generale, n. 65 del 18 marzo 2017) that sets out the conditions of deliverability relating to pharmacogenetic tests, limited to the analysis of *CYP2D6, CYP2C19* and *UGT1A1* combined with the drugs *gefitinib*, *atazanavir* and *erlotinib*, respectively (as specified in notes 94, 95, 96 of the same decree, Annexes 4 and 4D). Among these gene-drug pairs only CYP2C19-atazanavir appears to be consistent with some EMA recommendations reported in PharmGKB, especially for co-administration with *voriconazole*. It is unclear whether these notes were also taken into account in the publication of the new LEAs in August 2023.

Therefore, a contradictory and paradoxical picture emerges by comparing the provision of the Italian legislation, which remains somewhat unclear, with the recommendations of the EMA/AIFA, in particular regarding the necessity to offer guidelines on the prescriptive appropriateness of pharmacogenetic tests.

To address this topic, taking into account the latest scientific evidence, we assumed that the entire list of PGt indications obtained by merging the PREPARE and EMA reports, would be introduced in Italy. We have therefore compared this list with the new LEA statements to provide criteria for the appropriateness of prescribing the three PGt tests included and to extend the recommendations to the other genes included by the PREPARE study and EMA (Fig. 1).

The impact of this work is to emphasise that the recommendations to perform a pharmacogenetic test were approached differently by the PREPARE study and the EMA than by the new LEAs. The PREPARE study provides a set of gene-drug pairs for which clinical validity has been confirmed and the EMA notes the drugs with label pharmacogenetic testing indications. In contrast, there is no specific reference to drugs in the new LEAs and only three genes to be investigated with pharmacogenetic interest are mentioned (*CYP2D6, CYP2C19* and *UGT1A1*).

In conclusion, a problem of prescriptive appropriateness will be evident from the application of this new decree, as no targeted drug is mentioned. A useful result could be obtained by merging the PREPARE and EMA lists of drug-gene pairs. We propose to complete the list of genes for which pharmacogenetic testing is planned in Italy with the genes reported in the PREPARE study panel. To introduce prescriptive appropriateness criteria, a list of drugs for which these tests are indicated should be provided. Our proposal is therefore to take the gene-drug pairs presented by Swen and colleagues and extend them to include drugs for which there are EMA recommendations that should already be applied in Italy according to the prescriptive appropriateness decree.

### The roadmap of pharmacogenetics in Italy

The data presented here strongly supported the development of the following roadmap:

#### Translation and adoption of the DPWG and CPIC international guidelines

Given the clear relevance of the DPWG and CPIC guidelines, and the incorporation of these guidelines into PREPARE panel, it is justified to initiate the translation and adoption of these international guidelines in Italy. This process should consider the annotations provided by EMA/AIFA, which are particularly pertinent for the Italian healthcare system. This process should be conducted in close collaboration with other Italian scientific societies, including but not limited to the SIF. This cooperative effort is essential to ensure that the guidelines are effectively integrated into the Italian healthcare framework and that the recommendations align with the specific needs of the Italian population.

#### Elaboration of a dedicated informed consent to properly manage pharmacogenetics

The creation of a dedicated informed consent for managing pharmacogenetic information is essential. This consent should cover the handling of pharmacogenetic data generated by any test, including the potential discovery of secondary findings. Moreover, because pharmacogenetic tests may not carry the same as genetic tests used to diagnose diseases, there could be some flexibility during the prescribing process. This consent document should be readily available to individuals who, within the context of genetic analyses performed for various purposes, agree to allow the use of their genomic data for pharmacogenetic purposes. Furthermore, since pharmacogenetic tests may not have the same clinical weight as genetic tests used to diagnose diseases, there could be more flexibility during the prescribing process.

Despite this, in our opinion the post-test counselling should be exclusively conducted by geneticists who have the appropriate expertise to manage and interpret genomic data effectively. It is important to highlight that pharmacogenetic testing falls under the domain of geneticists, based on their specialized knowledge. Certainly, the clinician prescribing the medication can interpret the data in the context of the patient’s medication regimen.

#### Definition of minimum requirements for high-quality pharmacogenetic data

It is essential to define clear and specific minimum requirements for both the pharmacogenetic testing process and the subsequent data processing to ensure that the resulting pharmacogenetic data are of sufficient quality to be deemed “actionable.” This initiative aims to establish the baseline criteria to make pharmacogenetic data valuable and useful for clinical decision-making. These criteria should encompass not only the standards for the testing procedures but also the quality control and data processing steps. By defining these minimum requirements, the NHS can ensure that pharmacogenetic data are reliable, accurate, and clinically relevant for guiding patient care.

#### Identification of authorized personnel for gene testing and pharmacogenetic data interpretation

It is essential to clearly define and designate the individuals authorized to conduct gene testing and interpret pharmacogenetic data, irrespective of the data’s source. This aspect is crucial for standardizing the interpretation and communication of pharmacogenetic information to the individual owner, pharmacists, and healthcare providers. In this regard, only personnel with qualifications in germline testing, such as geneticists, should be granted the authority to perform pharmacogenetic evaluations. However, dialogue between the various scientific societies and the updating of training courses could make it possible to extend the skills required for the management of this type of germline test to other professionals, even beyond the prescription stage.

#### Creation of a ministerial working table to coordinate scientific societies (doctors, pharmacists) and AIFA

One of the biggest obstacles to the full implementation of pharmacogenetics in Italy is certainly the lack of coordination between the different actors, as demonstrated by the contradictions reported above. The most efficient way to overcome this issue is the creation of a ministerial discussion table involving representatives of the government, AIFA, EMA and the various scientific societies of geneticists and oncologists, as well as pharmacists.

## Conclusions

The field of pharmacogenetics holds great promise for the future of personalized medicines. As our understanding of the gene sequences that influence drug response deepens, the possibility of tailoring treatments to individual genetic profiles becomes increasingly apparent. The multiple benefits of genotype-specific therapy, which include reduced ADRs and increased clinical efficacy, underscore its importance in contemporary health care.

Based on the new LEAs, the Italian healthcare system is ready to embrace this paradigm shift, as evidenced by the recent introduction of additional genes in PGt testing, although prescriptive appropriateness is lacking and, for the same indications provided by EMA/AIFA, the number of loci is insufficient. It is therefore imperative to continuously refine and adapt pharmacogenetic guidelines in Italy as well, ensuring the safe and effective application of this innovative approach.

Nevertheless, the consensus on the usefulness of extended pharmacogenetic tests is not unanimous at the international level and the recommendations of various countries and consortia are only slightly overlapping. For this reason, in our opinion it is necessary to succeed in proposing at the national level to the relevant institutions a line of recommendations that clarifies the appropriateness and deliverability of this type of tests, to resolve the contradictions that have arisen since the approval of the new LEAs. This would help to bring Italian regulations in line with the latest scientific evidence on the usefulness and convenience of using pharmacogenetic testing, with the endpoint of more personalized and optimized therapy for patients.

### Electronic supplementary material

Below is the link to the electronic supplementary material.

Supplementary Material 1

Supplementary Material 2

## Data Availability

All data generated or analyzed during this study are included in this published article and its supplementary information files. Public data included in this study, comprising multiple drugs and clinical annotations, are available and adapted from the PharmGKB repository (https://www.pharmgkb.org/) and AIFA website (https://www.aifa.gov.it/), accessed on 15 January 2024.

## Abbreviations

EMA: European Medicines Agency
AIFA: Italian Medicines Agency
PGx: Pharmacogenomics
PGt: Pharmacogenetics
AIOM: Italian Association of Medical Oncology
SIF: Italian Society of Pharmacology
ADRs: Adverse Drug Reactions
FDA: Food and Drug Administration
NHSs: National Health Systems
DPWG: Dutch Pharmacogenetics Implementation Working Group
CPIC: Clinical Pharmacogenetics Implementation Consortium
LEA: Essential Levels of Care
SIGU: Italian Society of Human Genetics
OsMed: National Observatory on the Use of Medicines
