# Supplementary material for: Pharmacogenetics in Italy: current landscape and future prospects
Source: Hum Genomics. 2024 Jul 10;18:78. doi: 10.1186/s40246-024-00612-w (PMC11234611; doi:10.1186/s40246-024-00612-w)
Supplement: Supplementary file 2 — Supplementary Material 2 [file 40246_2024_612_MOESM2_ESM.docx]

PRIVACY REGULATION NOTES

Provision containing the prescriptions concerning the processing of special categories of data, pursuant to Article 21, paragraph 1 of Legislative Decree No. 101 of 10 August 2018.

For the purposes of this provision, the following are defined as:

(a) genetic data shall mean personal data relating to hereditary or acquired genetic characteristics of a natural person which provide unambiguous information on the physiology or health of that natural person, and which result in particular from the analysis of a biological sample of that natural person;

(b) pharmacogenetic test shall mean a genetic test designed to identify specific variations in the DNA sequence capable of predicting 'individual' response to drugs in terms of efficacy and relative risk of adverse events;

(c) pharmacogenomic testing: genetic testing aimed at the global study of variations in the genome or its products related to the discovery of new drugs and the further characterization of marketed drugs
